# Supplementary material for: Heat adaptation of phage T7 under an extended genetic code
Source: Virus Evol. 2021 Dec 1;7(2):veab100. doi: 10.1093/ve/veab100 (PMC8923235; doi:10.1093/ve/veab100)
Supplement: veab100_Supp [file veab100_supp.zip › Sequencing_Analysis.zip!/Sequencing_Analysis/A1/evidence/JC_SIDE_1_51.html]

BRESEQ :: Evidence


| New junction evidence | | | | | | | | | | | |
| --- | --- | --- | --- | --- | --- | --- | --- | --- | --- | --- | --- |
|  | | seq id | position | reads (cov) | reads (cov) | score | skew | freq | annotation | gene | product |
| \* | ? | NC\_001604.1 | 295 = | 3899 (1.260) | 25 (0.010) | 15/560 | 36.7 | 0.68% | intergenic (–/–) | *–/–* | –/– |
|  | ? | NC\_001604.1 | = 281 | 3655 (1.270) |  |  |  |  | intergenic (–/–) | *–/–* | –/– |
| Rejected: Coverage evenness skew score above cutoff. | | | | | | | | | | | |
| Rejected: Frequency below/above cutoff threshold. | | | | | | | | | | | |

Only 100 of 3922 total aligned reads displayed.  

`‑‑‑TCTCACAGTGTACGGACCTAAAGTTCCCCCATAGGGGGTACCTAAAGCCCAGCCAATCACCTAAAGTCAACCTTCGGTTGACCTTGAGGGTTCCCTAAGGGTTGGGGATGACCCTTGGGTTTGTCTTTGGGTGTTACCTTGAGTGTCTCTCTGTGTCCCTATCTGTTACAGTCTCCTAAAGTATCCTCCTAAAGTCACCTCCTAACGTCCATCCTAAAGCCAACACCTAAAGCCTACACCTAAAGACCCATCAAGTCAACGCCTATCTTAAAGTTTAAACATAAAGACC‑‑‑‑‑‑‑‑AGACCTAAAGACCAGACCTAAAGACACTACATAAAGACCAGACCTAAAGACGCCTTGTTGTTAGCCATAAAGTGATAACCTTTAATCATTGTCTTTATTAATACAACTCACTATAAGGAGAGACAACTTAAAGAGACTTAAAAGATTAATTTAAAATTTATCAAAAAGAGTATTGACTTAAAGTCTAACCTATAGGATACTTACAGCCATCGAGAGGGACACGGCGAATAGCCATCCCAATCGACACCGGGGTCAACCGGATAAGTAGACAGCCTGATAAGTCGCACGAAAAACAGGTATTGACA  >  NC_001604.1/1‑594`  

                                                                                                                                                                                                                                                                                                                 `|`                                                                                                                                                                                                                                                                                                             
`taagagacaaGTGTACTGACCTAAAGTTCCCCCATA‑GGGGTACCTAAAGCCCAGCCAATCACCTAAAGTCAACCTTCGGTTGACCTTGAGGGTTCCCTAAGGGTTGGGGATGACCCTTGGGTTTGTCTTTGGGTGTTACCTTGAGTGTCTCTCTGTGTCCCTATCTGTTACAGTCTCCTAAAGTATCCTCCTAAAGTCACCTCCTAACGTCCATCCTAAAGCCAACACCTAAAGCCTACACCTAAAGACCCATCAAGTCAACGCCTATCTTAAAGTTTAAACAtaaagacc‑‑‑‑‑‑‑‑agacctaaag                                                                                                                                                                                                                                                                                                         <  1:324752/292‑1 (MQ=255)`  
`aaagagacaggtgtACGGACCTAAAGTTCCCCCATA‑GGGGTACCTAAAGCCCAGCCAATCACCTAAAGTCAACCTTCGGTTGACCTTGAGGGTTCCCTAAGGGTTGGGGATGACCCTTGGGTTTGTCTTTGGGTGTTACCTTGAGTGTATCTCTGTGTCCCTATCTGTTACAGTCTCCTAAAGTATCCTCCTAAAGTCACCTCCTAACGTCCATCCTAAAGCCAACACCTAAAGCCTACACCTAAAGACCCATCAAGTCAACGCCTATCTTAAAGTTTAAACAtaaagacc‑‑‑‑‑‑‑‑agacctaaag                                                                                                                                                                                                                                                                                                         <  1:364717/291‑1 (MQ=255)`  
  `agagacaggtgtACTGACCTAAAGTTCCCCCATA‑GGGGTACCTAAAGCCCAGCCAATCACCTAAAGTCAACCTTCGGTTGACCTTGAGGGTTCCCTAAGGGTTTGGGATGACCCTTGGGTTTGTCTTTGGGTGTTACCTTGAGTGTCTCTCTGTGTCCCTATCTGTTACAGTCTCCTAAAGTATCCTCCTAAAGTCACCTCCTAACGTCCATCCTAAAGCCAACACCTAAAGCCTACACCTAAAGACCCATCAAGTCAACGCCTATCTTAAAGTTTAAACAtaaagacc‑‑‑‑‑‑‑‑agacctaaagac                                                                                                                                                                                                                                                                                                       <  1:153000/293‑1 (MQ=255)`  
  `aaagacaggtgtACGGACCTAAAGTTCCCCCATA‑GGGGTACCGAAAACCCAGCCAATATGCTAAATTCAACCTTCGGGTGACCTTGAGGGTTCCCAAAGGGTTGGGGATGACCCTTGGGTTTGTCTTTGGGTGTTACCTTGAGTGTCTCTCTGTGTCCCTATCCGTTACAGTCTCCTAAAGTATCCTCCTAAAGTCACCTCCTAACGTCCATCCTAAAGCCAACACCTAAAGCCTACACCTAAAGACCCATCAAGTCAACGCCTATCTTAAAGTTTAAACAtaaagacc‑‑‑‑‑‑‑‑agacctaaagac                                                                                                                                                                                                                                                                                                       <  1:242159/293‑1 (MQ=25)`  
   `gagacaggtgtACGGACCTAAAGTTCCCCCATA‑GGGGTACCTAAAGCCCAGCCAATCACCTAAAGTCAACCTTCGGTTGACCTTGAGGGTTCCCTAAGGGTTGGGGATGACCCTTGGGTTTGTCTTTGGGTGTTACCTTGAGTGTCTCTCTGTGTCCCTATCTGTTACAGTCTCCTAAAGTATCCTCCTAAAGTCACCTCCTAACGTCCATCCTAAAGCCAACACCTAAAGCCTACACCTAAAGACCCATCAAGTCAACGCCTATCTTAAAGTTTAAACAtaaagacc‑‑‑‑‑‑‑‑agacctaaagacc                                                                                                                                                                                                                                                                                                      <  1:414661/294‑1 (MQ=255)`  
   `aaggcaggggtACGGACCTAAAGTTCCCCCATA‑GGGGTACCTAAAGCCCAGCCAATCACCTAAAGTCAACCTTCGGTTGACCTTGAGGGTTCCCTAAGGGTTGTGGATGACCCTTGGGTTTGTCTTTGGGTGTTACCTTGAGTGTCTCTCTGTGTCCCTATCTGTTACAGTCTCCTAAAGTATCCTCCTAAAGTCACCTCCTAACGTCCATCCTAAAGCCAACACCTAAAGCCTACACCTAAAGACCCATCAAGTCAACGCCTATCTTAAAGTTTAAACAtaaagacc‑‑‑‑‑‑‑‑agacctaaagacc                                                                                                                                                                                                                                                                                                      <  1:124313/292‑1 (MQ=255)`  
          `gtgtATGGACCTAAAGTTCCCCCATA‑GGGGTACCTAAAGCCCAGCCAATCACCTAAAGTCAACCTTCGGTTGACCTTGAGGGTTCCCTAAGGGTTGGGGATGACCCTTGGGTTTGTCTTTGGGTGTTACCTTGAGTGTCTCTCTGTGTCCCTATCTGTTACAGTCTCCTAAAGTATCCTCCTAAAGTCACCTCATAACGTCCATCCTAAAGCCAACACCTAAAGCCTACACCTCAAGACCAATCAAGTCAACGCCTATCTTAACGTTTAAACCTAaagacc‑‑‑‑‑‑‑‑agacctaaagaccagacata                                                                                                                                                                                                                                                                                               >  1:135181/1‑301 (MQ=255)`  
          `gtgtACGGACCTAAAGTTCCCCCATA‑GGGGTACCTAAAGCCCAGTCAATCACCTAAAGTCAACCTTCGGTTGACCTTGAGGGTTCCCTAAGGGTTGGGGATGACCCTTGGGTTTGTCTTTGGGTGTTACCTTGAGTGTCTCTCTGTGTCCCTATCTGTTCCAGTCTCCTAAAGTATCCTCCTACAGTCACCTCCTAACGTCCATCCTAAAGCCAACACCTAAAGCCTCAACTTAAATACCCATCAAGTCAACGCCTATCTTAAAGTTTAAACATAaaaacc‑‑‑‑‑‑‑‑agacctaaagaccataccta                                                                                                                                                                                                                                                                                               >  1:469959/1‑301 (MQ=25)`  
          `gtgtACGGACCTAAAGTTCCCCCATA‑GGGGTACCTAAAGCCCAGCCAATCACCTAAAGTCAACCTTCGTTTGACCTTGAGGTTTCCCTAAGGGTTGCGTATGACCCTTGGGTTTGTCTTTGTGTGTTCCCTTGAGTGTCTCTCTGTGTCCCTCTCTGTTACAGTCTCCTAACTTATCCTCCTAAACTCACCTCCTCACGTCTATCCTATAGTCAACACCTAAAGCCTACACTCACAGACCCATCAAGTCATCGCCTATCTTAAAGTTTAAACATAaagacc‑‑‑‑‑‑‑‑acacctaacgtccagaccta                                                                                                                                                                                                                                                                                               >  1:300244/1‑301 (MQ=21)`  
          `gtgtACGGACCTAAAGTTCCCCCATA‑GGGGTACCTAAAGCCCAGCCAATCACCTAAAGTCAACCTTCGGTTGACCTTGAGGGTTCCCTAAGGGTTGGGGATGACGCTTTGGTTTGTCTTTGGGTGTTACCTTGAGTGTATCTCTGTGTCCCTATCTGTTACAGTCTCCTAAAGTATCCTCATAAAGTCACCTCCTAACGTCCATCCTAAAGCCAACACCTAAAGCCTACACCTAAAGACCCATCAAGTCAACGCCTATCTTAAAGTTTAAACAAaacgacc‑‑‑‑‑‑‑‑agacctaaagaacaaacctc                                                                                                                                                                                                                                                                                               >  1:115310/1‑300 (MQ=255)`  
          `gtgtACGGACCTAAAGTTCCCCCATA‑GGGGTACCTAAAGCCCAGCCAATCACCTAAAGTCAACCTTCGGTTGACCTTGAGGGTTCCCTAAGGGTTGGGGATGACCCTTGGGTTTGTCTTTGGGTGTTACCTTGAGTGTCTCTCTGTGTCCCTATCTGTTACAGTATCCTAAAGTATCCTCCTAAAGTCACCTCCTAACGTCCATCCTAAACCCAACACCTAAATCCTACACCTCACAACCCATCCAGTCAACACCTATCTTAAAGTTTAAACATAaagacc‑‑‑‑‑‑‑‑agccctaaagcccaaaacta                                                                                                                                                                                                                                                                                               >  1:465363/1‑301 (MQ=21)`  
          `gtgtACGGACCTAAAGTTCCCCCATA‑GGGGTACCTAAAGCCCAGCCAATCACCTAAAGTCAACCTTCGGTTGACCTTGAGGGTTCCCTAAGGGTTGGGGATGACCCTTGGGTTTGTCTTTGGCTGTTACCTTGAGTGCCTCTCTGTGTCCCTATCTGTTACAGTCTCCTAAAGTATCCTCCTAAAGTCACCTCCTAACGTCCATCCTAAAGCCAACACCTAAAGCCTACACCTAAAGACCCATCAAGTCAACGCCTATCTTAAAGTTTAAACATAaagacc‑‑‑‑‑‑‑‑agacctaaagaccagaccta                                                                                                                                                                                                                                                                                               >  1:388570/1‑301 (MQ=255)`  
          `gtgtACGGACCTAAAGTTCCCCCATA‑GGGGTACCTAAAGCCCAGCCAATCACCTAAAGTCAACCTTCGGTTGACCTTGAGGGTTCCATAAGGGTTGGGGATGACCCTTGGGTTTGTCTTTGGGTGTTACCTTGAGTGTCTCTCTGTGTCCCTATCTGTTACAGTCTCCTAAAGTATCCTCCTAAAGTCACCTCCTAACGTCCATCCTAAAGCCAACACCTAAAGCCTACACCTAAAGACCCATCAAGTCAACGCCTATCTTAAAGTTTAAACATAaagacc‑‑‑‑‑‑‑‑aaacctaaataccagaccta                                                                                                                                                                                                                                                                                               >  1:169723/1‑301 (MQ=255)`  
          `gtgtACGGACCTAAAGTTACCCCATA‑GGGGTACCTAAAGCCCAGCCAATCACCTAAAGTCAACCTTCGGTTGACCTTGAGGGTTCCCTAAGGGTTGGGGATGACCCTTGGGTTTGTCTTTGGGTGTTACCTTGAGTGTCTCTCTGTGTCCCTATCTGTTACAGTCTCCTAAAGTATCCTCCTAAAGTCACCTCCTAACGTCCATCCTAAAGCCAACACCTAAAGCCTACACCTAAAGACCCATCAAGTCAACGCCTATCTTAAAGTTTAAACAtaaagacc‑‑‑‑‑‑‑‑agacctaaagacctgtctct                                                                                                                                                                                                                                                                                               >  1:448068/1‑294 (MQ=255)`  
            `gtACGGACCTAAAGTTCCCCCATA‑GGGGTACCTAAAGCCCAGCCAATCACCTAAAGTCAACCTTCGGTTGACCTTGAGGGTTCCCTAAGGGTTGGGGATGACCCTTGGGTTTGTCTTTGGGTGTTACCTTGAGTGTCTCTCTGTGTCCCTATCTGTTACAGTCTACTAAAGTATCCTCCTAAAGTCACCTCCTAACGTCCATCCTAAAGCCAACACCTAAAGCCTACACCTAAAGACACATCACGTCAACGCCTATCTTAAAGTTTAAACATAAAgacc‑‑‑‑‑‑‑‑agacctaaagaccagacctaaa                                                                                                                                                                                                                                                                                             >  1:461012/1‑301 (MQ=255)`  
             `tACGGACCTAAAGTGCCCCCATA‑GGTGTACCTAAAGCCCAGCCAATCACCCAAACTCAACCTTCGGTGGACCTGGAGGGTTCCCTAAGGGTTGGGGATGACCCTGGGGTTTGTCTTTGGGTGTTACCTGGAGTGTCTCTCTGTGTCCCTATCTGTTACAGTCTCCTAAAGTATCCTACTAAAGCCACCTCCTAACGTCCATCCTAAAGCCAACACCAAAAGCCTACACCTAAAGACCCAGCAAGTAAACGCCTATCTTAAAGTTTAAACATAAAGacc‑‑‑‑‑‑‑‑agacctaaagaccagacctaaag                                                                                                                                                                                                                                                                                            <  1:491554/301‑1 (MQ=255)`  
                 `gACCTAAAGTTCCCCCATA‑GGGGTACCTAAAGCCCAGCCAATCACCTAAAGTCAACCTTCGGTTTACCTTGAGGGTTCCCTAAGGGTTGGGGATGACCCTTGGGTTTGTCTTTGGGTGTTACCTTGAGTGTCTCTCTGTGTCCCTATCTGTTACAGTCTCCTAAAGTATCCTCCTAAAGTCACCTCCTAACGTCCATCCTAAAGCCAACACCTAAAGCCTACACCTAAAGACCCATCAAGTCAACGCCTATCTTAAAGTTTAAACATAAAGACC‑‑‑‑‑‑‑‑AGACCTAAAGACCAGACCTAAAGacac                                                                                                                                                                                                                                                                                        <  1:291304/301‑1 (MQ=255)`  
                 `gACCTAAAGTTCCCCCATA‑GGGGTACCTAAAGCCCAGCCAATCACCTAAAGTCAACCTTCGGTTGACCTTGAGGGTTCCCTAAGGGTTGGGGATGACCCTTGGGTTTGTCTTTGGGTGTTACCTTGAGTGTCTCTCTGTGTCCCTATCTGTTACAGTCTCCTAAAGTATCCTCCTAAAGTCACCTCCTAACGTCCATCCTAAAGCCAACACCTAAAGCCTACACCTAAAGACCCATCAAGTCAACGCCTATCTTAAAGTTTAAACATAAAGACC‑‑‑‑‑‑‑‑AGACCTAAAGACCAGACCTAAAGaaac                                                                                                                                                                                                                                                                                        >  1:14769/1‑301 (MQ=255)`  
                 `gACCTAAAGTTCCCCCATA‑GGGGTACCTAAAGCCCAGCCAATCACCTAAAGTCAACCTTCGGTTGACCTTGAGGGTTCCCTAAGGGTTGGGGATGACCCTTGGGTTTGTCTTTGGGTGTTACCTTGAGTGTCTCTCTGTGTCCCTATCTGTTACAGTCTCCTAAAGTATCCTCCTAAAGTCACCTCCTAACGTCCATCCTAAAGCCAACACCTAAAGCCTACACCTAAAGACACATCAAGTAAACGCCTATCTTAAAGTTTAAACAtaaagacc‑‑‑‑‑‑‑‑agccctaaagctgtctcttatacacat                                                                                                                                                                                                                                                                                        >  1:220000/1‑284 (MQ=25)`  
                 `gACCTAAAGTTCCCCCATA‑GGGGTACCTAAAGCCCAGCCAATCACCTAAAGTCAACCTTCGGTTGACCTTGAGGGTTCCCTAAGGGTTGGGGATGACCCTTGGGTTTGTCTTTGGGTGTTACCTTGAGTGTCTCTCTGTGTCCCTATCTGTTACAGTCTCCT‑AAGTATCCTCCTAAAGTCACCTCCTAACGTCCATCCTAAAGCCAACACCTAAAGCCTACACCTAAAGACCCATCAAGTCAACGCCTATCTTAAAGTTTAAACATAAAGACC‑‑‑‑‑‑‑‑AGACCTAAAGACCAGACCTAAAGACACt                                                                                                                                                                                                                                                                                       >  1:166382/1‑301 (MQ=255)`  
                   `ccTAAAGTTCCCCCATA‑GGGGTACCTAAAGCCCAGCCAATCACCTAAAGTCAACCTTCGGTTGACCTTGAGGGTTCCCTAAGGGTTGGGGATGACCCTTGGGTTTGTCTTTGGGTGTTACCTTGAGTGTCTCTCTGTGTCCCTATCTGTTACAGTCTCCTAAAGTATCCTCCTAAAGTCACCTCCTAACGTCCATCCTAAAGCCAACACCTAAAGCCTACACCTAAAGACCCATCAAGTCAACGCCTATCTTAAAGTTTAAACATAAAGACC‑‑‑‑‑‑‑‑ACACCTAAAGACCAGACCTAAAGACACTa                                                                                                                                                                                                                                                                                      >  1:188359/1‑301 (MQ=255)`  
                        `gacagccccATA‑GGGGTACCTAAAGCCCAGCCCATCACCTAAAGTCAACCTTCGGTTGACCTTGAGGGTTCCCTAAGGGTTGGGGATGACCCTTGGGTTTGTCTTTGGGTGTTACCTTGAGTGTCTCTCTGTGTCCCTATCTGTTACAGTCTCCTAAAGTATCCTCCTAAAGTCACCTCCTAACGTCCATCCTAAAGCCAACACCTAAAGCCTACACCTAAAGACCCATCAAGTCAACGCCTATCTTAAAGTTTAAACATAAAGACC‑‑‑‑‑‑‑‑AGACCTAAAGACCAGACCTAAAGACACTACATaa                                                                                                                                                                                                                                                                                 <  1:431620/296‑1 (MQ=255)`  
                            `cccccATA‑GGAGTACCTACAGCCCAGCCAATCACCTAAAGTCAACCTTCTGTTGACCTTGAGGGTTTCCTAAGGGTTTGTGATGACCCTTGGGTTTGTCTTTGGGTTTTACCTTGAGTGTCTCTCTGTGTCCCTATCTGTTACAGTCTCCTATAGTATCCTCCTAAAGTCACCTCCTAACGTCCATCCTAAAGCCAACACCTAAAGCCTACACCTAAAGACCCATCAAGTCAATGCCTATCTTAAAGTTTAAACATAAAGACC‑‑‑‑‑‑‑‑AGACCTAAAGACCAGACCTAAAGACACTACATAAagac                                                                                                                                                                                                                                                                             <  1:354022/301‑1 (MQ=255)`  
                               `ccATA‑GGGGTACCTAAAGCCCAGCCAATCACCTAAAGTCAACCTTCGGTTGACCTTGAGGGTTCCCTAAGGGTTGGGGATGACCCTTGGGTTTGTCTTTGGGTGTTACCTTGAGTGTCTCTCTGTGTCCCTATCTGTTACAGTCTCCTAAAGTATCCTCCTAAAGTCACCTCCTAACGTCCATCCTAAAGCCAACACCTAAAGCCTACACCTAAAGACCCATCAAGTCAACGCCTATCTTAAAGTTTAAACATAAAGACC‑‑‑‑‑‑‑‑AGACCTAAAGACCAGACCTAAAGACACTACATAAagaccaa                                                                                                                                                                                                                                                                          >  1:247951/1‑300 (MQ=255)`  
                               `ccATA‑GGGGTACCTAAAGCCCAGCCAATCACCTAAAGTCAACCTTCGGCTGACCTTGAGGGTTCCCTAAGGTTTCGGGATGCCCCTTGTGTTTGTCTTTGGGTGTTACCTTGAGTTTCTCTCTGTGTCCCTCTCTGTTACAGTCTCCTCAATTTTCCTCCTACAGTCACCTCCTCACCTCCTTCCTAAAGCTAACACCTACCGCCTCCACCTAAAGACCCATCAATTCACCGCCTATCTTAAAGTTTCACCATACAGACC‑‑‑‑‑‑‑‑ATACCTAACGCCCAGACATACAGCCACtcactacagacctg                                                                                                                                                                                                                                                                          >  1:253644/1‑288 (MQ=18)`  
                                        `gTACCTAAAGTCCAGCCAATCTCCTAAAGTCAACCTTCGGTTGACCGTGAGGGTTCCATGAGGGTTGGGGATGACCCTTGGGTTTGTCTTTGGGTGTTACCTTGAGTGTCTCTCTGTGTCCCTATCTGTTACAGTCTCCTAAAGTATCCTCCTAAAGTCACCTCCTAACGTCCATCCTAAAGCCAACACCTAAAGCCTACACCTAAAGACCCATCAAGTCAACGCCTATCTTAAAGTTTAAACATAAAGACC‑‑‑‑‑‑‑‑AGACCTAAAGACCAGACCTAAAGACACTACATAAAGACCAGACCTAAAg                                                                                                                                                                                                                                                                  <  1:50186/301‑1 (MQ=255)`  
                                          `aCCTAAAGCCCAGCCAATCACCTAAAGTCAACCTTCGGTTGACCTTGAGGGTTCCCTAAGGGTTGGGGATGACCCTTGGGTTTGTCTTTGGGTGTTACATTGAGTGTCTCTCTGTGTCCCTATCTGTTACAGTCTCCTAAAGTATACTCCTAAAGTCACCTCCTAACGTCCATCCTAAAGCCAACACCTAAAGCCTACACCTAAAGACCCATCAAGTCAACGCCTATCTTAAAGTTTAAACATAAAGACC‑‑‑‑‑‑‑‑AGACCTAAGGACCAGACCTAAAGACACTACATAAAGACCAAACCTAAAGAc                                                                                                                                                                                                                                                                >  1:139165/1‑301 (MQ=255)`  
                                               `aaGCCCAGCCAATCACCTAAAGTCAACCTTCGGTTGACCTTGAGGGTTCCCTAAGGGTTGGGGATGACCCTTGGGTTTGTCTTAGGGTGTTACCTTGAGTGTCTCTTTGTGTCCCTATCTGTTACAGTCTCCTAAAGTATCCTCATAAAGTCACCTCCTAACGTCCATCCTAAAGCCAACACCTAAAGCCTACACCTAAAGACCCATCAAGTCAACGCCTATCTTAAAGTTTAAACATAAAGACC‑‑‑‑‑‑‑‑AGACCTAAAGACCAGACCTAAAGACACTACATAAAGACAAGACCTAAAGAcctgtc                                                                                                                                                                                                                                                           >  1:359780/1‑296 (MQ=255)`  
                                                        `tgtgtataagggacagaCCTTCGGTTGACCTTGAGGGTTCCCTAAGGGTTGGGGATGACCCTTGGGTTTGTCTTTGGGTGTTACCTTGAGTGTCTCTCTGTGTCCCTATCTGTTACAGTCTCCTAAAGTATCCTCCTAAAGTCACCTCCTAACGTCCATCCTAAAGCCAACACCTAAAGCCTACACCTAAAGACCCATCAAGTCAACGCCTATCTTAAAGTTTAAACATAAAGACC‑‑‑‑‑‑‑‑AGACCTAAAGACCAGACCTAAAGACACTACATAAAGACCAGACCTAAAGACGCCTTGTTGTTAGc                                                                                                                                                                                                                                                  <  1:107026/285‑1 (MQ=255)`  
                                                              `ccTAAAGTCAACCTTCGGTTGACCTTGAGGGTTCCCTAAGGGTTGGTGATGACCCTTGGGTTTGTCTTTGGGTGTTACCTTGAGTGTCTCTCTGTGTCCC‑ATCTGTTACAGTCTCCTAAAGTATCCTCCTAAAGTCACCTCCTAACGTCCATCCTAAAGACAACACCTAAAGCCTACACCTAAAGACCCATCAAGTCAACGCCTATCTTAAAGTTTAAACATAAAGACC‑‑‑‑‑‑‑‑AGCCCTAAAGACCAGACCTAAAGACACTACATAAAGACCAGACCTAAAGAAGCCTTGTTGTTAGCAATAAAg                                                                                                                                                                                                                                           >  1:275030/1‑301 (MQ=255)`  
                                                                              `ggTTGACCTTGAGGGATCCCTAAGGGTTGGGGATGACCCTTGGGTTTGTCTTTGGGTGTTACCTTGAGTGTCTCTCGGTGTCACTATCTGTTACAGGCTACTAAAGTATCCTCCTAAAGTCACCTCCTAACGTCCAGCCTAAAGCCAACACCTAAAGCCTACACCTAAAGACCAATCAAGTAAACGCCTATCTTAAAGTTTAAACATAAAGACC‑‑‑‑‑‑‑‑AGACCTAAAGACCAGACCTAAAGACACTACAGAAAGACCAGACCTAAAGACGCCTTGTTGTTAGCCATAAAGTGATAACCTTTAATc                                                                                                                                                                                                                            <  1:57682/301‑1 (MQ=255)`  
                                                                              `atgtgtataagagacagcccTAAGGGTTTGGGATGACCCTTGGGTTTGTCTTTGGGTGTTACCTTGAGTCTCTCTCTGTGTCCCTATCTGTTACAGTCTCCTAAAGTATCCTCCTAAAGTCACCTCCTAACGTCCATCCTAAAGCCAACACCTAAAGCCTACACCTAAAGACCCATCAAGTCAACGCCTATCTTAAAGTTTAAACATAAAGACC‑‑‑‑‑‑‑‑AGACCTAAAGACCAGACCTAAAGACACTACATAAAGACCAGACCTAAAGACGCCTTGTTGTTAGCCATAAAGTGATAACCTTTAATc                                                                                                                                                                                                                            <  1:90776/284‑1 (MQ=255)`  
                                                                                          `gggTTCCCTAAGGGTTGGGGATGACCCTTGGGTTTGTCTTTGGGTGTTACCTTGAGTGTCTCTCTGTGTCCCTATCTGTTACAGTCTCCTAAAGTATCATCCTAAAGTCACCTCCTAACGTCCATCCTAAAGCCAACACCTAAAGCCTACACCTAAAGACCCATCAAGTCAACGCCTATCTTAAAGTTTAAACATAAAGACC‑‑‑‑‑‑‑‑AGACCTAAAGACCAGACCTAAAGACACTACATAAAGACCAGACCTAAAGACGCCTTGTTGTTAGCCATAAAGTGATAACCTTTAATCATTGTCTttatt                                                                                                                                                                                                                <  1:78081/301‑1 (MQ=255)`  
                                                                                               `cccacaGGGGTGGGGATTACCCTTTGGCTTTTCTTTTGGTGTTACCTTTAGTGTCTTTCTGTGTACCTAACCTTTACAGTCTCCTAAAGTCTCCTCCTAAAGTCACCTCCTAACGTCCATCCTAAAGGCAACACCTAAAGCCTACACCTAAAGACACATCAAGTCAACGCCTATCTTAAAGTTTAAACATAAAGACC‑‑‑‑‑‑‑‑AGACCTAAAGACCAGACCTAAAGACACTACCTAAAGACCAGACCTAAAGACGCCTTGTTGTTAGCCATAAAGTGATAACCTTTAATCATTGTCTTTATTAATAc                                                                                                                                                                                                           <  1:456871/296‑1 (MQ=255)`  
                                                                                               `cccTAAGGGTTGGGGATTACCCTTGGGTTTGTCTTTGGGTGTTACCTTGAGTGTCTCTCTGTGTCCCTATCTGTTACAGTCTCCTAAAGTATCCTCCTAAAGTCACCTCCTAACGTCCATCCTAAAGCCAACACCTAAAGCCTACACCTAAAGACCCATCAAGTCAACGCCTGTCTTAAAGTTTAAACATAAAGACC‑‑‑‑‑‑‑‑AGACCTAAAGACCAGACCTAAAGACACTACATAAAGACCAGACCTAAAGACGCCTTGTTGTTAGCCATAAAGTGATCACCTTTAATCATTTTCTTTATTAATAc                                                                                                                                                                                                           >  1:21625/1‑301 (MQ=255)`  
                                                                                               `cccTAAGGGTTGGGGATGACCCTTGGGTTTGTCTTTGGGTGTTACCTTGAGTGTCTCTCTGTGTCCCTATCTGTTACAGTCTCCTAAAGTATCCTCCTAAAGTCACCTCCTAACGTCCATCCTAAAGCCAACACCTAAAGCCTACACCTAAAGACCCATCAAGTCAACGCCTATCTTAAAGTTTAAACATAAAGACC‑‑‑‑‑‑‑‑AGACCTAAAGACCAGACCTAAAGACACTACATAAAGACCAGACCTAAAGACGCCTTGTTGTTAGCCATAAAGTGATAACCTTTAATCATTGTCTTTATTAATAc                                                                                                                                                                                                           <  1:10439/301‑1 (MQ=255)`  
                                                                                               `cccTAAGGGTTGGGGATGACCCTTGGGTTTGTCTTTGGGTGTTACCTTGAGTGTCTCTCTGTGTCCCTATCTGTTACAGTCTCCTAAAGTATCCTCCTAAAGTCACCTCCTAACGTCCATCCTAAAGCCAACACCTAAAGCCTACACCTAAAGACCCATCAAGTCAACGCCTATCTTAAAGTTTAAACATAAAGACC‑‑‑‑‑‑‑‑AGACCTAAAGACCAGACCTAAAGACACTACATAAAGACCAGACCTAAAGACGCCTTGTTGTTAGCCATAAAGTGATAACCTTTAATCATTGTCTTTATTAATAc                                                                                                                                                                                                           <  1:174688/301‑1 (MQ=255)`  
                                                                                                      `ggTTGGGGATGACCCTTGGGTTTGTCTTTGTGTGTTACCTTGAGTGTCTCTCTGTGTCCCTATCTGTTACAGTCTCCTAAAGTATCCTCCTAAAGTCACCTCCTAACGTCCATCCTAAAGCCAACACCTAAAGCCTACACCTAAAGACCCATCAAGTCAACGCCTATCTTAAAGTTTAAACATAAAGACC‑‑‑‑‑‑‑‑AGACCTAAAGACCAGACCTAAAGACACTACATAAAGACCAGACCTAAAGACGCCTTGTTGTTAGCCATAAAGTGATAACCTTTAATCATTGTCTTTATTAATACAACTCAc                                                                                                                                                                                                    <  1:62705/301‑1 (MQ=255)`  
                                                                                                          `ggggATGACCCTTGGGTTTGTCTTTGGGTGTTACCTTGAGTGTCTCTCTGTGTCCCTATCTGTTACAGTCTCCTAAAGTATCCTCCTAAAGTCACCTCCTAACGTACATACTAAAGCCAACACCTAAAGCCTACACCTAAAGACCCATCAAGTCAACGCCTATCTTAAAGTTTAAACATAAAGACC‑‑‑‑‑‑‑‑AGACCTAAAGACCAGACCTAAAGACACTACATAAAGACCAGACCTAAAGACGCCTTGTTGTTAGCCATAAAGTGATAACCTTTAATAATTGTCTTTATTAATACAACTCACtata                                                                                                                                                                                                >  1:31473/1‑301 (MQ=255)`  
                                                                                                           `gggATGACCCTTGGGTTTGTCTTTGGGTGTTACCTTGAGTGTCTCTCTGTGTCCCTATCTGTTACAGTCTCCTAAAGTATCCTCCTAAAGTCACCTCCTAACGTCCATCCTAAAGCCAACACCTAAAGCCTACACCTAAAGACCCATCAAGTCAACGCCTATCTTAAAGTTTAAACATAAAGACC‑‑‑‑‑‑‑‑AGACCTACAGACCATACCTAAAGACACTACATAAAGACCAGACCTAAAGACGCCTTGTTGTTAGCCATAAAGTGATAACCTTTAATCATTATCTTTATtactacctgtctctttta                                                                                                                                                                                               >  1:345513/1‑289 (MQ=255)`  
                                                                                                             `gacaggccTTGGGTTTGTCTTTGGGTGTTACCTTGAGTGTCTCTCTGTGTCCCTATCTGTTACAGTCTCCTAAAGTATCCTCCTAAAGTCACCTCCTAACGTCCATCCTAAAGCCAACACCTAAAGCCTACACCTAAAGACCCATCAAGTCAACGCCTATCTTAAAGTTTAAACATAAAGACC‑‑‑‑‑‑‑‑AGACATAAAGACCAGACCTAAAGACACTACATAAAGACCAGACCTAAAGACGCCTTGTTGTTAGCCATAAAGTGATAACCTTTAATCATTGTATTTATTAATACAACTCACTATAAgg                                                                                                                                                                                             <  1:452831/295‑1 (MQ=255)`  
                                                                                                             `gaagACCCTTGGGATTGTCTTTGAGTGGTTCCATGAGAGTCCCTCATTATCCCTAACTGTTACAGTCACCTAAAGTATCCTCCTAAAGTCAACTCAGAACGTCCAGCCTAAAGCAAAAACCTAAAGCCTACACCTAAAGACCCATCAAGTCAACACCTATCTTAAAGTGTAAACATAAAGACC‑‑‑‑‑‑‑‑AGACCTAAAGACCAGACCTAAAGACACTACATAAAGACCAGACATAAAGACGCCTTGTTGTTAGCCATAAAGTGATAACCTTGAATCATTGTCTTTATTAATACAACTCACTATAAgg                                                                                                                                                                                             <  1:497599/298‑1 (MQ=255)`  
                                                                                                             `gATTACCCTTGGGTTTGTCTGTGGGTGTTACCTTGAGTGTCTCTCTGTGTCCCTATCTGTTACAGTCTCCTAAAGTATCCTCCTAAAGTCACCACCTAACATACAGCCTAAAGCCAACACCAAAAGCCTACACCTAAAGACCCATCAAGTCAACGCATATCTTAAAGTTTAAACATAAAGACC‑‑‑‑‑‑‑‑AGACCTAAAGACCAGACCTAAAGACACTACATAAAGACCAGACCTAAAGACGCCTTGTTGTTAGCCATAAAGTGATAACCTTTAATCATTGTCTTTATTAATACAACTCACTATAAgg                                                                                                                                                                                             <  1:229788/301‑1 (MQ=255)`  
                                                                                                             `acagACCCTTGGGTTTGTCTTTGGGTGTTACCTTGAGTGTCTCTCTGTGTCCCTATCTGTTACAGTCTCCTAAAGTATCCTCCTAAAGTCACCTCCTAACGTCCATCCTAAAGCCAACACCTAAAGCCTACACCTAAAGACCCATCAAGTCAACGCCTATCTTAAAGTTTAAACATAAAGACC‑‑‑‑‑‑‑‑AGACCTAAAGACCAGACCTAAAGACACTACATAAAGACCAGACCTAAAGACGCCTTGTTGTTAGCCATAAAGTGATAACCTTTAATCATTGTCTTTATTAATACAACTCACTATAAgg                                                                                                                                                                                             <  1:184262/298‑1 (MQ=255)`  
                                                                                                                  `cccTTGGGTTTGTCTTTGGGTGTTACCTTGAGTGTCTCTCTGTGTCCCTATCTGTTACAGTCTCCTAAAGTATCCTCCTAAAGTCACCTCCTAACGTCCATCCTAAAGCCAACACCTAAAGCCTACACCTAACGACCCATCAAGTCAACGCCTATCTTAAAGTTTAAACATAAAGACC‑‑‑‑‑‑‑‑AGACCTAAAGACCAGACCTAAAGACACTACATAAAGACCAGACCTAAAGACGCCTTGTTGTTAGCCATAAAGTGATAACCTTTAATCATTTTCTTTATTACTACAACTCACTATAAGtagaga                                                                                                                                                                                        >  1:161627/1‑301 (MQ=255)`  
                                                                                                                  `cccTTGGGTTTGTCTTTGGGTGTTACCTTGAGTGTCTCTCTGTGTCCCTATCTGTTACAGTCTCCTAAAGTATCCTCCTAAAGTCACCTCCTAACGTCCATCCTAAAGCCAACACCTAAAGCCTACACCTAAAGACCCATCAAGTCAACGCCTATCTTAAAGTTTAAACATAAAGACA‑‑‑‑‑‑‑‑AGACCTAAAGACAAGACCTAAAGACACTACATAAAGACCAGACCTAAAGACGCCTTGTTGTTAGCCATAAAGTGATAACCTTTAATCATTGTCTTTATTAATAcctgtctcttatacacatct                                                                                                                                                                                        >  1:87391/1‑282 (MQ=255)`  
                                                                                                                     `ttGGGTTTGTCTTTGGGTGTTACCTTGGGGGTTTCTCTGTGTCCCTATCTGTTACAGTCTCCTAAAGTATCCTCCTAAAGTCACCTCCAAACGTCCATCCTAAAGCCAACACCTAAAGCCTACACCTAAAGAACCATCAAGTCAACGCCTATCTTAAAGTTTAAACATAAAGACC‑‑‑‑‑‑‑‑AGACCTAAAGACCAGACCTAAAGACACTACATAAAGACCAGACCTAAAGACGCCTTGTTGTTAGCCATAAAGTGATAACCTTTAATCATTGTCTTTATTAATACAACTCACTATAAGGAGAGACaa                                                                                                                                                                                     <  1:478239/301‑1 (MQ=255)`  
                                                                                                                       `gggTTTGTCTTTGGGTGTTACCTTGAGTGTCTCTCTGTGTCCCTATCTGTTACAGTCTCCTAAAGTATCCTCCTAAAGTCACCTCCTAACGTCCATCCTAAAGCCAACACCTAAAGCCTACACCTAAAGACCCATCAAGTCAACGCCTATCTTAAAGTTTAAACATAAAGACC‑‑‑‑‑‑‑‑AGACCTAAAGACCAGACCTAAAGACACTACATAAAGAACAGACCTAAAGACGCCTTGTTGTTAGCCATAAAGTTATAACCTTTAATCATTGTCTTTATTAATACAACTCACTATAAGGAGAGACAACt                                                                                                                                                                                   >  1:295324/1‑301 (MQ=255)`  
                                                                                                                           `ttGTCTTTGGGTGTTACCTTAGATGTCTCTCTGTGTCCCTATCTGTTACAGTCTCCTAAAGTATCCTACTGAAGTCACCTCCTAACGTCCATCCTAAAGCCAACACCTAAAGCCTACACCTAAAGACCCATCAAGTCAACGCCTATCGTAAAGTTTAAACTTAAAGACCAGACCTAAAGACCTAAAGACCAGACCTAAAGACACTACATAAAGACCAGACCTAAATACGCCTTGTTGTTAGCCATAAAGTGATAACCTTTAATCATTGTCTTTATTAATACAACTCACTATAAGGAGAGAc                                                                                                                                                                                       <  1:378555/301‑1 (MQ=255)`  
                                                                                                                            `tGTCTTTGGGTGTTACCTTGAGTGTCTCTATGTGTCCCTATCTGTTACAGTCTACTAAAGTATCCGCCTAAAGTCACCTCCTAACGTCCATCCTAAAGCCAACACCTAAAGCCTACACCTAAAGAACCATCAAGTCAACGCCTATCTTAAAGTTTAAACATAAAGACC‑‑‑‑‑‑‑‑AGACCTAAAGAACAGACCTAAAGACACTACATAAAGACCAGACCTAAAGACGCCTTGTTGTTAGACATAAAGTGATAACCTTTAATCATTGTCTTTATTAATACAACTCACTATAAGGAGAGACAACTTAAag                                                                                                                                                                              <  1:486906/301‑1 (MQ=255)`  
                                                                                                                             `gTCTTTGGGTGTTACCTTGAGTGTCTCTCTGTGTCCCTATCTGTTACAGTCTCCTAAAGTATCCTCCTAAAGTCACCTCCTAACGTCCATCCTAAAGCCAACACCTAAAGCCTACACCTAAAGACCCATCAAGTCAACGCCTATCTTAAAGTTTAAACATAAAGACC‑‑‑‑‑‑‑‑AGACCTAAAGACCAGACCTAAAGCAACTACATAAAGACCAGACCTAAAGACGCCTTGTTGTTAGCCATAAAGTGATAACCTTTAATCATTGTCTTTATTAATCAAACTAACTATAAGGAGAGCCAAATTAAaga                                                                                                                                                                             >  1:400944/1‑301 (MQ=255)`  
                                                                                                                             `gTCTTTGGGTGTTACCTTGAGTGTCTCTCTGTGTCCCTATCTGTTACAGTCTCCTAAAGTATCCTCCTAAAGTCACCTCCTAACGTCCATCCTAAACCCAACACCTAAAGCCTACACCTAAATACCCATCAAGTCAACGCCTATCTTAAAGTTTAAACATAAAGACC‑‑‑‑‑‑‑‑AGACCTAAAGACCAGACCTAAAGACACTACATAAAGACCAGACCTAAAGACGCCTTGTTGTTAGCCATAAAGTGATAACCTTTAATCATTGTCTTTATTAATACAACTCACTATAAGgactgtctcttatacac                                                                                                                                                                             >  1:481602/1‑286 (MQ=255)`  
                                                                                                                             `gTCTTTGGGTGTTACCTTGAGTGTCTCTCTGTGTCCCTATCTGTTACAGTCTCCTAAAGTATCCTCCTAAAGTCAACTCCTAACGTCCATCCTAAAGCCAACACCTAAAGCCTACACCTAAAGACCCATCAAGTCAACGCCTATCTTAAAGTTTAAACATAACGACC‑‑‑‑‑‑‑‑AGACCTAAAGAACAGACCTAAAGACACTACATACAGACCAGATCTAAAAACGCCTTGTCGTTAGCTATACAGTAATAACCTTTACTCATTGTCTTTATTAATACACCTCACTATAAggctgtcacatatccaaa                                                                                                                                                                             >  1:422/1‑285 (MQ=255)`  
                                                                                                                              `gtgtataatagacagttGAGTGGCTCCCTGTGTCCCTATCTGTTACAGTCTCCTAAAGTATCCTCCTAAAGTAACATCCTAACGTCCATCCTAAAGCCAACACCTAAAGCCTACACCTAAAGACCCATCAAGTCAACGCATATCTTAAAGTTTAAACATAAAGACC‑‑‑‑‑‑‑‑AGAACTAAAGACCAGACCTAAAGACACTACATAAAGACCAGACCTAAAGACGCCTTGTTGTTAGCCATAAAGTGATAACCTATAATCATTGTCTTTATTAATACAACTCACTAAAAGGAGAGACAACTTAAagag                                                                                                                                                                            <  1:304431/286‑1 (MQ=255)`  
                                                                                                                              `gacaggggTGTTACCTTGAGTTTCTCTCTGTGTCCCTATCTGTTACAGTCTCCTAAAGGATCCTCCTAAAGTCACCTCCTAACGTCCATCCTAAAGCCAACACCTAAAGCCTACACCTAAAGACCCATCAAGTCAACGCCTATCTTAAAGTTTAAACATAAAGACC‑‑‑‑‑‑‑‑AGACCTAAAGACCAGACCTAAAGACACTACATAAAGACCAGACCTAAAGACGCCTTGTTGTTAGCCATAAAGTGATAACCTTTAATCATTGTCTTTATTAATACAACTCACTATAAGGAGAGACAACTTAAagag                                                                                                                                                                            <  1:39658/296‑1 (MQ=255)`  
                                                                                                                                       `tgtgtataagagacagctctGTGTCCCTATCTGTTACAGTCTCCTAAAGTATCCTCCTAAAGTCACCTCCTAACGTCCATCCTAAAGCCAACACCTAAAGCCTACACCTAAAGACCCATCAAGTCAACGCCTATCTTAAAGTTTAAACATAAAGACC‑‑‑‑‑‑‑‑AGACCTAAAGACCAGACCTAAAGACACTACATAAAGACCAGACCTAAAGACGCCTTGTTGTTAGCCATAAAGTGATAACCTTTAATCATTGTCTTTATTAATACAACTCACTATAAGGAGAGACAACTTAAAGAGACTTAAAAg                                                                                                                                                                   <  1:40868/285‑1 (MQ=255)`  
                                                                                                                                               `gAGTGTCTCTCTGTGTCCCTATCTGTTACAGTCTCCTAAAGTACCCTCCTAAAGTCACCTCCTAACGTCCATCCTAAAGCCAACACCTAAAGCCTACACCTAAAGACCCATCAAGTCAACGCCTATCTTAAAGTTTAAACATAAAGACC‑‑‑‑‑‑‑‑AGACCTAAAGACCAGACCTAAAGACACTACATAAAGACCAGACCTAAAGACGCCTTGTTGTTAGCCATAAAGTGATAACCTTTAATCATTGTCTTTATTAATACAACTCACTATAAGGAGAGACAACTTAAAGAGACTTAAAAGATTAAttt                                                                                                                                                           >  1:404215/1‑301 (MQ=255)`  
                                                                                                                                                 `gtgtCTCTCTGTGTCCCTATCTGTTACAGTCTCCTAAAGTATCCTCCTAAAGTCACCTACTAACGTCAATCCTAAAGCCAACACCTAAAGTCTACACCTAAAGACCCATCAAGTCAACGCCTATCATAAAGTTTAAACATAAAGACC‑‑‑‑‑‑‑‑AGACCTAACGACCAGATCTAAAGACACTACATAAAGACCAGACCTAAAGAAGCCTTATTGTTAGCCATAAAGTGATAAACTTTAATCATTGTCTTAATTAATAAAACTCACTATAAGGAGAGACAACTTAAAGAGACTTAAAAGATTAATTTaa                                                                                                                                                         >  1:262526/1‑301 (MQ=255)`  
                                                                                                                                                       `ctctGTGTCCCTATCTGTTACAGTCTCCTAAAGTATCCTCCTAAAGTCACCTCCTAACGTCCATCCTAAAGCCAACACCTAAAGCCTACACCTAAAGACCCATCAAGTCAACGCCTATCTTAAAGTTTAAACATAAAGACC‑‑‑‑‑‑‑‑AGACCTAAAGACCAGACCTAAAGACACTACATAAAGACCAGACCTAAAGACGCCTTGTTGTTAGCCATAAAGTGATAACCTTTAATCATTGTCTTTATTAATACAACTCACTATAAGGAGAGACAACTTAAAGAGACTTAAAAgctgtctcttatacaca                                                                                                                                                   >  1:19263/1‑285 (MQ=255)`  
                                                                                                                                                         `ctttgtCCCGATCTGTTACAGTCTCCTAAAGTATCCTCCTAAAGTCACCTCCTAACGTCCATCCTAAAGCCAACACCCAAAGCCTACACCTAAAGACCCATCAAGTCAACGCCTATCTTAAAGTTTAAACATAAAGACC‑‑‑‑‑‑‑‑AGACCTAAAGACCAGACCTAAAGACACTACATAAAGACCAGACCTAAAGACGCCTTGTTGTTAGCCATAAAGTGATCACCTTTAATCATTGTCGTTATTAATACAACTCACTATAAGGAGAGACAACTTAAAGAGACTTAAAAGATTAATTTAAAATTGATc                                                                                                                                                 <  1:201259/298‑1 (MQ=255)`  
                                                                                                                                                                     `cTGTTACAGTCTCCTAAAGTATCCTCCTAAAGTCACCTCCTAACGTCCATCCTAAAGCCAACACCTAAAGCCTACACCTAAAGACCCATCAAGTCAACGCCTATCTTAAAGTTTAAACATAAAGACC‑‑‑‑‑‑‑‑AGACCTAAAGACCAGACCTAAAGACACTACATAAAGACCAGACCTAAAGACGACTTGTTGTTAGCCATAAAGTGATAACCTTTAATCATTGTCTTTATTAATACAACTCACTATAAGGAGTGACAAATTAAAGAGACTTAAAAGATTAATTTAAAATTTATCAAAAAGAGTAtt                                                                                                                                     >  1:349957/1‑301 (MQ=255)`  
                                                                                                                                                                       `ggagatgtgtaaaagagacagctcctAAAGTCACCTCCTAACGTCCATCCTAAAGCCAACACCTAAAGCCTACACCTAAAGACCCATCAAGTCAACGCCTATCTTAAAGTTTAAACATAAAGACC‑‑‑‑‑‑‑‑AGACCTAAAGACCAGACCTAAAGACACTACATAAAGACCAGACCTAAAGACGCCTTGTTGTTAG‑CATAAAGTGATAACCTTTAATCATTGTCTTTATTAATACAACTCACTATAAGGAGAGACAACTTAAAGAGACTTAAAAGATTAATTTAAAATTTATCAAAAAGAGTATGGAc                                                                                                                                  <  1:383772/280‑1 (MQ=255)`  
                                                                                                                                                                       `gTTACAGTCTCCTAAAGTATCCTCCTAAAGTCACCTCCTAACGTCCATCCTAAAGCCAACACCTAAAGCCTAAAACTAAAGACACATCAAGTCAACGATTATCTTAAAGTTTAAACATAAAGACA‑‑‑‑‑‑‑‑AGACCTAAAGACCAGACCTAAAGACACTACATACAGACCAGCACTAAAGACGCCTTGTTGTTAGCCATAAAGTGAAACCCTTTAATAATTGACTTTATTAATACAACTCACTATAAGAAGAGACAACTTAAAGAAACTTAAAAGATACATAAAAAATTTATACAAAAGATAATTGa                                                                                                                                   >  1:95324/1‑301 (MQ=255)`  
                                                                                                                                                                              `agatgtgtataagagacagaaaGTCACCTCCTAACGTCCATCCTAAAGCCAACACCTAAAGACTACACACAAAGACCAATCAAGTCAAAGCCTATCACAAAGTTTAAAAATAAAGACC‑‑‑‑‑‑‑‑AGACCTAAAGACCAGACCTAAAGACACTACATAAAGACCAGACCTAAAGACGCCTTGTTGTTAGCCATAAAGTGATAACCTTTAATCATTGTCTTTATTAATACAACTCACTATAAGGAGAGACAACTTAAAGAGACTTAAAAGATTAATTTAAAATTTATCAAAAAGAGTATTGACTTAAAg                                                                                                                            <  1:225102/282‑1 (MQ=255)`  
                                                                                                                                                                               `ctcCTAAAGTATCCTCCTAAAGTCACCTCCTAACGTCCATCCTAAAGCCAACACCTAAAGCCTACACCTAAAGACCCATCACTTCAACGCCTATCTTAAAGTTTAAACATAAAGATC‑‑‑‑‑‑‑‑AGACCTAACGACCAGACCTAAAGACACTACATAAAGACCAGACCTAAAGACGCCTTGTTGTTAGCCATAAAGTTATAACCTTTAATCATTGTCTTTATTAATACAACTTACACTAAGTAGAGACAACTTAAATAGACTTAAAAGTTTAATTTAAAATCTATCAAAAAGAGTATTGACTTaatgc                                                                                                                           >  1:438045/1‑298 (MQ=255)`  
                                                                                                                                                                               `ctcCTAAAGTATCCTCCTAAAGTCACCTCCTAACGTCCATCCTAAAGCCAACACCTAAAGACTACACCTAAAGACCCATCAAGTCAACGCCTATCTTAAAGTTTAAACATAAAGACC‑‑‑‑‑‑‑‑AGACCTAAAGACCAGACCTAAAGACACTACATAAAGACCAGACCTAAAGACGCCTTGTTGTTAGCCATAAAGTGATAACCATTAATCATTGTCTTTATTAATACAACTCACTATAAGGAGAGACAACTTAAAGAGACTTAAAAGATTAATTTAAAATTTATAAAAAAGATTATTAAATTAAAga                                                                                                                           >  1:368649/1‑300 (MQ=255)`  
                                                                                                                                                                                `tcCGAAAGTATCCTCCTAAAGTCACCTCCTAACGTCCATCCTAAAGCCAACACCTAAAGCCTACACCTAAAGAACCATCAAGTCAACGCCTATCTTAAAGTTTAAACATAAAGACC‑‑‑‑‑‑‑‑AGACCTAAAGACCAGACCTAAAGACACTACATAAAGACCAGACCTAAAGACGCCTTGTTGTTAGCCATAAAGTGATAACCTTTAATCATTGTCTTTATTAATACAACTCACTATAAGGAGAGACAACTTAAAGAGACTTAAAAGATTAATTTAAAATTTATCAAAAAGAGTATTGACTTAAAGTc                                                                                                                          <  1:257580/301‑1 (MQ=255)`  
                                                                                                                                                                                `cccTAAAGTATCCTCCTAAAGTCCCCTCCTAACGTCCATCCTAAAGCCAACACCTAAAGCCTACACCTAAAGACCCATCAAGTCAACGCCTATCTTAAAGTTTAAACATAAATACC‑‑‑‑‑‑‑‑AGACCTAAAGACCAGACCTAAAGACACTACATAAAGACCAGACCTAAAGACGCCTTGTTGTTAGCCATAAAGTGATAACCTTTAATCATTGTCTTTATTAATACAACTCACTATAAGGAGAGACAACTTAAAGAGACTTAAAAGATTAATTTAAAATTTATCAAAAAGAGTATTGACTTAAAGTc                                                                                                                          <  1:270978/300‑1 (MQ=255)`  
                                                                                                                                                                                          `acctcctAGAGTCACCTCCTAACGTCCATCCCAAAGCCAACACCTAAAGCCTACACCTAAAGACCCATCAAGTCAACGACTATCTTAAAGTTTAAACATAAAGAAC‑‑‑‑‑‑‑‑AGACCTAAAGACCAGACCTAAAGACACTACATAAAGACCAGACCTAAAGACGCATTGGTGTTAGCCATAAAGTGATAACCTTTAATCATTGTCTTTATTAATACAACTCACTATAAGGAGAGACAACTTAAAGAGACTTAAAAGATTAATTTAAAATTTATCAAAAAGAGTATTGACTTAAAGTCTAACCTATAg                                                                                                                <  1:238776/300‑1 (MQ=255)`  
                                                                                                                                                                                           `cctcctAAAGTCACCTCCTAACGTCCATCCTAAAGCCAACACCTAAAGCCTACACCTAAAGACCCATCAAGTCAACGCCTATCTTAAAGTTTAAACATAAAGACC‑‑‑‑‑‑‑‑AGACCTAAAGACCAGACCTAAAGACACTACATAAAGACCAGACCTAAAGACGCCTTGTTGTTAGCCATAAAGTGATAACCTTTAATCATTGTCTTTATTAATACAACTCACTATAAGGAGAGACAACTTAAAGAGACTTAAAAGATTAATTTAAAATTTATCAAAAAGAGTATTGACTTAAAGTCTAACCTATAgg                                                                                                               >  1:144643/1‑301 (MQ=255)`  
                                                                                                                                                                                            `ctcctAAAGTCACCTCCTAACGTCCATCCTAAAGCCAACACCTAAAGCCTACACCTAAAGACCCATCAAGTCAACGCCTATCTTAAAGTTTAAACATAAAGACC‑‑‑‑‑‑‑‑AGACCTAAAGACCAGACCTAAAGACACTACATAAAGACCAGACCTAAAGACGCCTTGTTGTTAGCCATAAAGTGATAACCTTTAATCATTGTCTTTATTAATACAACTAACTTTAAGGAGAGACAACTTAAAGAGACTTAAAAGATTAATTTAAAATTTATCAAAAAGAGTATTGACTTAATGTCTAACATATAGGa                                                                                                              >  1:73025/1‑301 (MQ=255)`  
                                                                                                                                                                                             `tcctAGAGTCACCGACTAACGTCCATCCTAAAGCCAACACCTAAAGCCTACACCTAAAGGCACATTAAGTCAACGCATATCTTAAAGTTTAAACATAAAGACC‑‑‑‑‑‑‑‑AGACCTAAAGACCAGACATAAAGACACTACATAAAGAACAGACCTAAAGACGCCTTGTTGGTAGCCATAGAGTGATAACCTTTAATCATTGTCTTTATTAATACAACTAACTATAAGGAGAGACAACTTAAAGAGACTTAAAAGATTAATGTAAAATTTATCAAAAAGAGTATTGACTTAAAGTCTAACCTATAGGAt                                                                                                             <  1:233718/301‑1 (MQ=255)`  
                                                                                                                                                                                             `ccctAAAGTCACCTCCTCACGTCTATCCTAAAGCCAACACCTAAAGCCTACACCTAAAGAACCATCAAGTCAACGCCTATCTTAAAGTTTAAACATAAAGACC‑‑‑‑‑‑‑‑AGACCTAAAGACCAGACCTAAAGACACTACATAAAGACCAGACCTAAAGACGCCTTGTTGTTAGCCATAAAGTGATAACCTTTAATCATTGTCTTTATTAATACAACTCACTATAAGGAGAGACAACTTAAAGAGACTTAAAAGATTAATTTAAAATTTATCAAAAAGAGTATTGACTTAAAGTCTAACCTATAGGAt                                                                                                             <  1:156912/300‑1 (MQ=255)`  
                                                                                                                                                                                              `cctAAAGTCACCTCCTAACGTCCATCCTAAAGCCAACACCTAAAGCCTACACCTAAAGACCCATCAAGTCAACGCCTATCTTAAAGTTTAAACATAAAGACC‑‑‑‑‑‑‑‑AGACCTAAAGACCAGACCTAAAGACACTACATAAAGACCAGACCTAAAGACGCCTTGTTGTTAGCCATAAAGTGATAACCTTTAATCATTGTATTTATTAATACAACTCACTATAAGGAGAGACAACTTAAAGAGACTTAAAAGATTAATTTAAAATTTATCAAAAAGAGTATTGACTTAAAGTCTAAccctgtctctt                                                                                                            >  1:82971/1‑292 (MQ=255)`  
                                                                                                                                                                                                  `aaGTCACCTCCTAACGTCCATCCTAAAGCCAACACCTAAAGCCTACACCTAAAGACCCATCAAGTCAACGCCTATCTTAAAGTTTAAACATAAAGACC‑‑‑‑‑‑‑‑AGACCTAAAGACCAGACCTAAAGACACTACATAAAGACCAGACCTAAAGACGCCTTGTTGTTAGCCATAAAGTGATAACCTTTAATCATTGTCTTTATTAATACAACTCACTATAAGGAGAGACAACTTAAAGAGACTTAAAAGATTAATTTAAAATTTATCAAAAAGAGTATTGACTTAAAGTCTAACCTATAGGATACTTa                                                                                                        <  1:3409/301‑1 (MQ=255)`  
                                                                                                                                                                                                   `gagacagtcctAACGTCCATCCTAAATCCAACACCTAAATCCTACACCTAAAGACCCATCAAGTCAACGCCTATCTTAAAGTTTAAACATAAAGACC‑‑‑‑‑‑‑‑AGACCTAAAGACCAGACCTAAAGACACTACATAAAGACCAGACCTAAAGACGCCTTGTTGTTAGCCATAAAGTGATAACCTTTAATCATTGTCTTTATTAATACAACTCACTATAAGGAGAGACAACTTAAAGAGACTTAAAAGATTAATTTAAAATTTATCAAAAAGAGTATTGACTTAAAGTCTAACCTATAGGATACTTAc                                                                                                       <  1:427630/294‑1 (MQ=255)`  
                                                                                                                                                                                                   `aGTCACCTCCTAACGTCCAACCTAAAGCCAACACCTAAAGCCTACACCTAAAGACCCATCAAGTCAACGCCTATCTTAAAGTTTAAACATAAAGACC‑‑‑‑‑‑‑‑AGACCTAAAGACCAGACCTAAAGACACTACATAAAGACCAGACCTAAAGACGCCTTGTTGTTAGCCATAAAGTGATAACCTTTAATCATTGTCTTTATTAATACAACTCACGATAAGGAGAGACAACTTAAAGAGACTTAAAAGATTAATTTAAAATTTATCAAAAAGAGTATTGACTTAAAGTCTAACCTATAGGATACTTAc                                                                                                       <  1:309316/301‑1 (MQ=255)`  
                                                                                                                                                                                                       `gaagatgtgtatacgagacagaGCCAACACCTAAAGCCTACACCTAAACACCCATCAAGTCAATGCCTATCTTAAAGTTTAAACATAAAGACC‑‑‑‑‑‑‑‑AGACCTAAAGACCAGACCTAAAGTCACTACATAAAGTCCAGACCTACAGACGCCTTGTTGTTAGCCATAAAGTTATAACCTTTAATCATTGTCTTTATTAATACAACTCACTATAAGGAGAGACAACTTAAAGAGACTTAAAAGATTAATTTAAAATTTATCAAAAAGAGTATTGACTTAAAGTCTAACCTATAGGATACTTACAGcc                                                                                                   <  1:282740/280‑1 (MQ=255)`  
                                                                                                                                                                                                       `agctcctAACGTCCATCCTAAAGCAAACACCTAAAGCCTACACCTAAAGACCCATCAAGTAAACGCCTATCTTAAAGTTTAAACATAAAGACC‑‑‑‑‑‑‑‑AGACCTAAAGACCAGACCTAAAGACACTACATAAAGACCAGACCTAAAGACGCCTTGTTGTTAGCCATAAAGTGATAACCTTTAATCATTGTCTTTATTAATACAACTAACTATAAGGAGAGACAACTTAAAGAGACTTAAAAGATTAATTTAAAATTTATCAAAAAGAGTATTGACTTAAAGTCTAACCTATAGGATACTTACAGcc                                                                                                   <  1:119296/299‑1 (MQ=255)`  
                                                                                                                                                                                                        `cctcctAACGTCCATCCTAAAGCCAACACCTAAAGCCTACACCTAAAGACCCATCAAGTCAACGCCTATCTTAAAGTTTAAACATAAAGACC‑‑‑‑‑‑‑‑AGACCTAAAGACCAGACCCAAAGACACTACATAAAGACCAGACCTAAAGACGCCTTGTTGTTAGCCATAAAGTGATAACCTTTAATCATTGTCTTTATTAATACAACTCACTATAAGGAGAGACAACTTAAAGAGACTTAAAAGATTAATTTAAAATTTATCAAAAAGAGTATTGACTTAAAGTCTAACCTATAgctgtctcttataca                                                                                                  >  1:442910/1‑287 (MQ=255)`  
                                                                                                                                                                                                         `ctcctAACGTCCATCCTAAAGCCAACACCTAAAGCCTACACCTAAAGACCCATCAAGTCAACGCCTATCTTAAAGTTTAAACATAAAGACC‑‑‑‑‑‑‑‑AGACCTAAAGACCAGACCTAAAGACACTACATAAAGACAAGCCCTAAAGCCGCCTTTTTGTTAGCCATAAAGTGATAACCTTTAATCATTGTCTTTATTAATACAACTCACTATAAGGAGAGACAACTTAAAAAGACTTAAAAGATTAATTTAAAATTTATCAAAAAGAGTATTGACTTAAAGTCTAACCTATAGAATACTTActtgtct                                                                                                 >  1:267304/1‑295 (MQ=255)`  
                                                                                                                                                                                                               `aCGTCCATCCTAAAGCCAAATCCTAAAGCCTACACCTAAAGACCCATCAAGTCAACGCCTATCATAAAGTTTAAACATAAAGACC‑‑‑‑‑‑‑‑AGACCTAAATACCATACCTAAAGACACTACATAAAGAACAGTCCTAAAGACGCCTTGTTGTTAGCCATACAGTGATAACCTGTAATCATTGTCTTTATTAATACAACTCACTATAAGGAGAGACATCTTAAAGAGACTTAAAAGATTAATTTAAAATTTATCAAAAAGAGTATTGACTTAAAGTCTAACCTATAGGATACTTACAGCCATCGagag                                                                                           <  1:67352/301‑1 (MQ=255)`  
                                                                                                                                                                                                                `cctCCAACCTAAAGCCAACACCAAAAGCCTACACCTACAGACCCATCAAGTCAACGCCTATATTAAAGTTTAAACAAAAAGAAC‑‑‑‑‑‑‑‑AGACCTAAAGACCAAACCTAAAGACACTACATAAAGACCAGACCTAAAGACGCCTTTTTGTTAGCCATAAAGTGATAACCTTTAATCATTGTCTTTATTAATTCAACTCACTATAAGGAGAGACAACTTAAAGAGACTTAAAAGATTAATTTAAAATTTATCAAAAAGAGTATTGACTTAAAGTCTAACCTATAGGATACTTACAGCCATCGAGAgg                                                                                          <  1:319681/299‑1 (MQ=255)`  
                                                                                                                                                                                                                                    `ccTAAAGCCTACACCTAAAGACCCATCAAGTCAACGCCTATCTTAAAGTTTAAACATAAAGACC‑‑‑‑‑‑‑‑AGACCTAAAGACCAGACCTAAAGACACTACATAAAGACCAGACCTAAAGACGCCTTGTTGTTAGCCATAAAGTGATAACCTTTAATCATTGTCTTTATTAATACAACTCACTATAAGGAGAGACAACTTAAAGAGACTTAAAAGATTAATTTAAAATTTATCAAAAAGAGTATTGACTTAAAGTCTAACCTATAGGATACTTACAGCCATCGAGATGGACACGGCGAATAGCCATcc                                                                      >  1:373735/1‑301 (MQ=255)`  
                                                                                                                                                                                                                                     `cTAAAGCCTACACCTAAAGACCCATCAAGTCAACGCCTATCTTAAAGTTTAAACATAAAGACC‑‑‑‑‑‑‑‑AGACCTAAAGACCAGACCTAAAGACACTACATAAAGACCAGACCTAAAGACGCCTTGTTGTTAGCCATAAAGTGATAACCTTTAATCATTGTCTTTATTAATACAACTCACTATAAGGAGAGACAACTTAAAGAGACTTAAAAGATTAATTTAAAATTTATCAAAAAGAGTATTGACTTAAAGTCTAACCTATAGGATACTTACAGCCATCGAGAGGGACACGGCGAATAGCCATccc                                                                     <  1:196478/301‑1 (MQ=255)`  
                                                                                                                                                                                                                                       `agagacagcacCTAAAGACCCATCAAGTCAACGCCTATCTTAAAGTTTAAACATAAAGACC‑‑‑‑‑‑‑‑AGACCTAAAGACCAGACCTAAAGACACTACATAAAGACCAGACCTAAAGACGCCTTGTTGTTAGCCATAAAGTGATAACCTTTAATCATTGTCTTTATTAATACAACTCACTATAAGGAGAGACAACTTAAAGAGACTTAAAAGATTAATTTAAAATTTATCAAAAAGAGTATTGACTTAAAGTCTAACCTATAGGATACTTACAGCCATCGAGAGGGACACGGCGAATAGCCATCCCaa                                                                   <  1:337689/293‑1 (MQ=255)`  
                                                                                                                                                                                                                                               `cacCTAAAGACCCATCAAGTCAACGCCTATCTTAAAGTTTAAACATAAAGACC‑‑‑‑‑‑‑‑AGACCTAAAGACCAGACCTAAAGACACTACATAAAGACCAGACCTAAAGACGCCTTGTTGTTAGCCATAAAGTGATAACCTTTAATCATTGTCTTTATTAATACAACTCACTATAAGGAGAGACAACTTAAAGAGACTTAAAAGATTAATTTAAAATTTATCAAAAAGAGTATTGACTTAAAGTCTAACCTATAGGATACTTACAGCCATCGAGAGGGACACGGCGAATAGCCATCCCAATCGACAcc                                                           >  1:205889/1‑301 (MQ=255)`  
                                                                                                                                                                                                                                               `cacCTAAAGACCCATCAAGTCAACGCCTATCTTAAAGTTTAAACATAAAGACC‑‑‑‑‑‑‑‑AGACCTAAAGACCAGACCTAAAGACACTACATAAAGACCAGACCTAAAGACGCCTTGTTGTTAGCCATAAAGTGATAACCTTTAATCATTGTCTTTATTAATAAAACTCACTATAAGGAGAGACAACTTAAAGAGACTTAAAAGATTAATTTAAAATTTATCAAAAAGAGTATTGAGTTAAAGTCTAACGTATAGGTTACTTAAAGCCATCGAGGGGGACACGGCGAATAACCATCCCAATCGACAcc                                                           >  1:100062/1‑301 (MQ=255)`  
                                                                                                                                                                                                                                                 `ccTAAAGACCCATCAAGTCAACGCCTATCTTAAAGTTTAAACATAAAGACC‑‑‑‑‑‑‑‑AGACCTAAAGACCAGACCTAAAGACACTACATAAAGACCAGACCTAAAGACGCCTTGTTGTTAGCCATAAAGTGATAACCTTTAATCATTGTCTTTATTAATACAACTCACTATAAGGAGAGACAACTTAAAGTGACTTAAAAGATTAATTTAAAATTTATCAAAAAGAGTATTGACTTAAAGTCTAACCTATAGGATACTTACAGCCATCGAGAGGGACACGGCGAATAGCCATCCCAATCGACACCgg                                                         >  1:506074/1‑301 (MQ=255)`  
                                                                                                                                                                                                                                                 `ccTAAAGACCCATCAAGTCAACGCCTATCTTAAAGTTTAAACATAAAGACC‑‑‑‑‑‑‑‑AGACCTAAAGACCAGACCTAAAGACACTACATAAAGACCAGACCTAAAGACGCCTTGTTGTTAGCCATAAAGTGATAACCTTTAATCATTGTCTTTATTAATACAACTCACTATAAGGAGAGACAACTTAAAGAGACTTAAAAGATTAATTTAAAATTTATCAAAAAGAGTATTGACTTAAAGTCTAACCTATAGGATACTTACAGCCATCGAGAGGGAAACGGCGAATAGCCATCCAAATCGACACCgg                                                         >  1:111299/1‑301 (MQ=255)`  
                                                                                                                                                                                                                                                                `agtacaCGCCTATCTTAAAGTTTAAACATAAAGACC‑‑‑‑‑‑‑‑AGACCTAAATACCAGACCTAAAGACACTACATAAAGACCAGACCTAAAGACGCCTTGTTGTTAGCCATAAAGTGATAACCTTTAATCATTGTCTTTATTAATACAACTCACTATAAGGAGAGACAACTTAAAGAGACTTAAAAGATTAATTTAAAATTTATCAAAAAGAGTATTGACTTAAAGTCTAACCTATAGGATACTTACAGCCATCGAGAGGGACACGGCGAATAGCCATCCCAATCGACACCGGGGTCAACCGGATAAg                                          <  1:278792/296‑1 (MQ=255)`  
                                                                                                                                                                                                                                                                 `gTCAACGCCTATCTTAAAGTTTAAACATAAAGACC‑‑‑‑‑‑‑‑AGACCTAAAGACCAGACCTAAAGACACTACATAAAGACCAGACCTAAAGACGCCTTGTTGTTAGCCATAAAGTGATAACCTTTAATCATTGTCTTTATTAATACAACTCACTATAAGGAGAGACAACTTAAAGAGACTTAAAAGATTAATTTAAAATTTATCAAAAAGAGTATTGACTTAAAGTCTAACCTATAGGATACTTACAGCCATCGAGAGGGACACGGCGAATAGCCATCCCAATCGACACCGGGGTCAACCGGATAAGt                                         >  1:211919/1‑301 (MQ=255)`  
                                                                                                                                                                                                                                                                    `aaCGCCTATCTTAAAGTTTAAACATAAAGACC‑‑‑‑‑‑‑‑AGACCTAAAGACCAGACCTAAAGACACTACATAAAGACCAGACCTAAAGACGCCTTGTTGTTAGCCATAAAGTGATAACCTTTAATCATTGTCTTTATTAATACAACTCACTATAAGGAGAGACAACTTAAAGAGACTTAAAAGATTAATTTAAAATTTATC‑AAAAGAGTATTGACTTAAAGTCTAACCTATAGGATACTTACAGCCATCGAGAGGGACACGGCGAATAGCCATCCCAATCGACACCGGGGTCAACCGGATAAGTAGAc                                     <  1:286697/301‑1 (MQ=255)`  
                                                                                                                                                                                                                                                                           `aTCTTAAAGTTTAAACATAAAGACC‑‑‑‑‑‑‑‑AGACCTAAAGACCAGACCTAAAGACACTACATAAAGACCAGACCTAAAGACGCCTTGTTGTTAGCCATAAAGTGATAACCTTTAATCATTGTCTTTATTAATACAACTCACTATAAGGAGAGACAACTTAAAGAGACTTAAAAGATTAATTTAAAATTTATCAAAAAGAGTATTGACTTAAAGTCTAACCTATAGGATACTTACAGCCATCGAGAGGGACACGGCGAATAGCCATCCCAATCGACACCGGGGTCAACCGGATAAGTATACAGCCTg                               >  1:473650/1‑301 (MQ=255)`  
                                                                                                                                                                                                                                                                           `aTCTTAAAGTTTAAACATAAAGACC‑‑‑‑‑‑‑‑AGACCTAAAGACCAGACCTAAAGACACTACATAAAGACCAGACCTAAAGACGCCTTGTTGTTAGCCATAAAGTGATAACCTTTAATCATTGTCTTTATTAATACAACTCACTATAAGGAGAGACAACTTAAAGAGACTTAAAAGATTAATTTAAAATTTATCAAAAAGAGTATTGACTTAAAGTCTAACCTATAGGATACTTACAGCCATCGAGAGGGACACGGCGAATAGCCATCCCAATCGACACCGGGGTCAACCGGATAAGTAGACAGCCTg                               >  1:392577/1‑301 (MQ=255)`  
                                                                                                                                                                                                                                                                               `aaaaTTTTAAACATAAAGAAC‑‑‑‑‑‑‑‑AGACCTAAATACCAGACCTAAAGACACTACATGAAGACCAGACCTAAAGACGCCTTGTTGTTAGCCATAAAGTGATAACCGTTAATTATTGTATTTATTAATACAACTCACTATAAGGAGAGACAACTTAAAGAGACTTAAAAGATTAATTTAAAATTTGTCAAAAAGAGTATTGACTTAAAGTCTAACCTATAGGATACTTACAGCCATCGAGAGGGACACTGCGAATAGCCATCCCAATCGACACCGGGGTCAACCGTATAAGTAGACAGCCTGATaa                           <  1:52487/300‑1 (MQ=255)`  
                                                                                                                                                                                                                                                                                                             `gacctaaagaccagacctaaagacACTACTTAAAGACCAGCCCTAAAGACGACTTGTTGTTAGCCATAAAGTGATAACCTTTAATCATTGTCTTTATTATTACAACTCACTATAAGGAGAGACACCTTAAAGCGACTCAAAAGATTAATTTAAAATTTCTCAAAAAGAGTATTGACTTAAAGTCTAACCTATAGGATACTTACAGCCATCGAGAGGGACACGGCGAATAGCCATCCCAATCGACACCGGGGTCAACCGGATAAGTAGACAACCTGATAAGTCGctgtctcttatacacatt     >  1:179187/1‑284 (MQ=255)`  
                                                                                                                                                                                                                                                                                                               `cctaaagaccagacctaaagacACTACATAAAGACCAGACCTAAAGACGCCTTGTTGTTAGCCATAAAGTGATAACCTTTAATCATTGTCTTTATTAATACAACTCACTATAAGGAGAGACAACTTAAAGAGACTTAAAAGATTAATTTAAAATTTATCAAAAAGAGTATTGACTTAAAGTCTAACCTATAGGATACTTACAGCCATCGAGAGGGACACGGCGAATAGCCATCCCAATCGACACCGGGGTCAACCGGATAAGTAGACAGCCTGATAAGTCGCACGAAAAACAGGTATTGac   >  1:13042/1‑301 (MQ=255)`  
                                                                                                                                                                                                                                                                                                               `cctaaagaccagacctaaagacACTACATAAAGACCAGACCTAAAGACGCCTTGTTGTTAGCCATAAAGTGATAACCTTTAATCATTGTCTTTATTAATACAACTCACTATAAGGAGAGACAACTTAAAGAGACTTAAAAGATTAATTTAAAATTTATCAAAAAGAGTATTGACTTAAAGTCTAACCTATAGGATACTTACAGCCATCGAGAGGGACAAGGCGAATAGCCATCCCAATAGACACCGGGGTCAACCGGATAAGTAGACAGCCTTATAAGTCGCACGAAAAACAGATATTgct   >  1:333715/1‑299 (MQ=255)`  
                                                                                                                                                                                                                                                                                                                `ctaaagaccagacctaaagacACTACATAAAGACCAGACCTAAAGACGCCTTGTTGTTAGCCATAAAGTGATAACCTTTAATCATTGTCTTTATTAATACAACTCACTATAAGGAGAGACAACTTAAAGAGACTTAAAAGATTAATTTAAAATTTATCAAAAAGAGTATTGACTTAAAGTCTAACCTATAGGATACTTACAGCCATCGAGAGGGACACGGCGAATAGCAATCCCAATCGACACCGGGGTCAACCAGATGAGTATACAGCCTGATAAGTCGCACGAAAAACAGGTATTGaca  >  1:329428/1‑301 (MQ=255)`  

                                                                                                                                                                                                                                                                                                                 `|`                                                                                                                                                                                                                                                                                                             
`‑‑‑TCTCACAGTGTACGGACCTAAAGTTCCCCCATAGGGGGTACCTAAAGCCCAGCCAATCACCTAAAGTCAACCTTCGGTTGACCTTGAGGGTTCCCTAAGGGTTGGGGATGACCCTTGGGTTTGTCTTTGGGTGTTACCTTGAGTGTCTCTCTGTGTCCCTATCTGTTACAGTCTCCTAAAGTATCCTCCTAAAGTCACCTCCTAACGTCCATCCTAAAGCCAACACCTAAAGCCTACACCTAAAGACCCATCAAGTCAACGCCTATCTTAAAGTTTAAACATAAAGACC‑‑‑‑‑‑‑‑AGACCTAAAGACCAGACCTAAAGACACTACATAAAGACCAGACCTAAAGACGCCTTGTTGTTAGCCATAAAGTGATAACCTTTAATCATTGTCTTTATTAATACAACTCACTATAAGGAGAGACAACTTAAAGAGACTTAAAAGATTAATTTAAAATTTATCAAAAAGAGTATTGACTTAAAGTCTAACCTATAGGATACTTACAGCCATCGAGAGGGACACGGCGAATAGCCATCCCAATCGACACCGGGGTCAACCGGATAAGTAGACAGCCTGATAAGTCGCACGAAAAACAGGTATTGACA  >  NC_001604.1/1‑594`

  

Alignment Legend

Aligned base mismatch/match (shaded by quality score): `ATCG/ATCG` `< 3 ≤` `ATCG/ATCG` `< 10 ≤` `ATCG/ATCG` `< 19 ≤` `ATCG/ATCG` `< 33 ≤` `ATCG/ATCG` `< 38 ≤` `ATCG/ATCG`

Unaligned base: `atcg`    Masked matching base: `atcg`    Alignment gap: `‑`    Deleted base: `‑`

Reads not counted as support for junction

read\_name Not counted due to insufficient overlap past the breakpoint.

read\_name Not counted due to not crossing MOB target site duplication.
